# Editorial

**Published:** 1915-08

**Authors:** 


### EDITORIAL

The Atlanta Journal-Record of Medicine has its Business Office at 23 West Alabama Street where all business communications should be sent.

Remittances are payable to The Journal-Record of Medicine.

Concerning Original Communications and Editorial matters, address Richard R. Daly, M. D., 708 Empire Life Building, Atlanta, Ga.

Reprints of Original Articles will be furnished at cost price. Requests for them had best be made on the Manuscript.

Upon request, we will present, postpaid, to each contributor of an original article, twenty copies of The Journal-Record of Medicine containing such article.

### THE HARRISON LAW.

In the article we present by Mr. Hooper Alexander, U. S. District Attorney for the Northern District of Georgia, we have an analysis of the law that should be of value to every reader. Mr. Alexander is in closest sympathy with the profession in what he knows is its endeavor to comply with the law and its intention to support it and he shows clearly that there is no antagonism between the law and the legitimate practice of medicine. Not only that but there is no intent to advise the doctor how he should practice. The law expresses the determination of the people at large to do protective work in the field of the narcotic habit and to weed out the awful fruitage there.

The measure was made primarily a revenue one so that it could reach every part of the country uniformly and be within the limits of the Constitution of the United States, but it yields no cash revenue. The cost of its enforcement in money is likely always to exceed the income. The benefits derived from it will be in men and women.

After his talk in Atlanta before the Fulton County Medical Society, measures were at once taken to care for the habitues of drugs who could not be suddenly denied their portions. The City Detention and Isolation Hospital where small-pox has been treated, was empty and there was ample space in the cottages and extensive grounds where these patients could be kept and cared for if they desired. It was offered by the city and has been made use of successfully during the last month.

The cure of the opium and cocaine habits is conditional upon the absence of opportunity to use the drug for a long time after the immediate and pressing symptoms of the poisoning have been eliminated. It is hoped that because of the restrictions that the law imposes upon the sale of these drugs and the increasing severity with which they are enforced, a man can safely be freed from hospital supervision in a much shorter time than has formerly been consumed in attempting to restore his will-power. This hope can be given to the victim as well as enjoyed by the physician if time proves it a reliable one.

When we can say to a man who seems almost too degraded for any salvation that there is a chance for his recovery and then something even better to keep him well, we shall know that whatever work we have to do or personal annoyance we have to withstand in connection with the operation of this law, was of no account in comparison with the fine result. Then there is the protection of the young men and women, too, who are constantly acquiring the habit under modifications of vice that come with other new things. If the sale of these habit-forming drugs were even more restricted than it is, the results would make it all worth while.

The experience in Jacksonville, Fla. has been that when habitues were offered public treatment and told that they could recover and be protected by the law from getting any more dope, the majority of them secured money from friends and took suitable private treatment which up to then had seemed useless. We commend this fact to the thoughtful attention of any community which seems to be faced with the impossible financial problem of caring for its habitues. That they must be cared for in some way goes without saying. Their sufferings during the craving stage are too intense to be disregarded. Yet no man wants to treat them symptomatically and become a complacent prescriber which is really a pander. The community must do it and the community is responsible because it has licensed the sale of the drugs.

It can be done and done more easily than was expected provided always there is an uncompromising enforcement of the law and a prompt and severe punishment of every purveyor of the interdicted drugs.

---

### PELLAGRA.

In the New Orleans Medical and Surgical Journal for September, Dr. E. M. Perdue, of Kansas City, Mo., advances in an exhaustive thesis a new cause and a new treatment for pellagra. He shows that the distribution of the disease is the same as that of clay soils and the absense of "hard" drinking water. This is further explained by the presence of a colloidal silica in solution or suspension in the drinking water and he states that "pellagra is a chronic intoxication due to colloidal silica." This silica is extracted from clay by the water of the wells and rivers. The symptom of acidosis is caused by the storing of the silicates in the system, absorbing the water and setting the hydrochloric acid free. The treatment is simple and consists first in drinking hard water, made so with lime if necessary, and then injecting a 10 per cent. solution of sodium citrate every day or every other day. Of course, symptoms are to allayed by appropriate attention, but with the fundamental principle carried out cases are cured in from 30 to 60 days.

The author does not give statistics of cases, but presents his ideas for trial by the medical world. Nothing could be fairer.

Pellagra remains a mystery despite all the theories that our best men have advanced. Each has approximated the truth and some of them so nearly that we have thought the discovery was at hand, but there is still incertitude. It is elusive and alluring. It seems to disprove itself and then to show the pathway along which there is no escape for it if diligently pursued. The reward for its actual definition and the discovery of its cure would satisfy any man, for he would confer a world-wide blessing and his renown and fame would endow his family. We add this possible cause to the list in the hope of encouraging its investigation.

---

### SOLDIERS AND DISEASE.

It is a surprising statement of statistics compiled by the English, that the Germans have thus far lost but 15,800 men by disease in the war.

Just what this proportion to the size of the army is we can not say exactly, but is is so small as to attract the attention of all who are interested in sanitation and hygiene.

The memories we have of the Spanish war are already growing dim in many ways, but the one that stands out strongly and which is in the way of recruiting the National Guard is the great death rate we had in camps and among soldiers who never saw the front; while the care of those who were doing the fighting reflected no credit upon those responsible for the methods.

If there is need of preparedness in war matters at all that need is paramount to all others in the care of the soldier which will make him a good one. And all the training in the world that drill and fighting information can give will not make a good soldier of a sick man or of an unnecessarily tired and dirty one.

The ideas of sanitation that are good in the army are of value anywhere and everywhere. Only when they control life immediately as they do in the army, we seem to think them of greater importance. Municipalities are arising to the control of certain conditions and are co-operating with the Marine Service in many ways. All this is fine, but it should have rapid fruition in the training given in the schools where discipline is already a matter of instruction, and the young people should have ingrained upon them a respect for that sort of control.

In the present warring armies, the doctors have an authority never before reached, and the result is that they issue orders that are obeyed rather than make requests that are considered in a few days by some higher and unsympathetic, possibly political, authority. They take the responsibility and thus they can deliver the goods in sound men and serviceable soldiers.

We are not at war and no one wants to be, but we should be prepared for this activity. The best preparation is not the theoretical one of the regimental drill hall or encampment, but the every day work of sanitation and hygienic discipline in each community no matter how small. There are problems of health to be solved wherever two or three are gathered together and the opportunity demands recognition from every intelligent physician right at his own door yard where he can teach his neighbors and guide his community the way of health.

---

### TWILIGHT SLEEP.

Once more this subject comes to our attention and this time brings what might have been promised when it got to the stage of magazine reading article advertising and newspaper argument. Many of the men who were competent to make investigations by reason of extensive hospital facilities have discarded it. The Jewish Maternity and Lebanon Hospitals and a part at least of Bellevue in New York now administer it only when it is asked for by the patients and they are not requesting it when they realize that delirium is likely to follow or be connected with it. It is true that there are some men who remain enthusiastic concerning it, but they are urging the construction of an institution for that purpose. We can not help feeling that there is an association somehow between such an institution and those that are maintained for diseases where advertising, though ethical in character, is needed and that the practice will be in some measure commercialized. It is a pretty narrow specialty after all, and it is likely to go that route.

The fact remains, so far as our observation goes, that the method is not practical in everyday obstetrics such as the general practitioner has to meet. He says so, himself, though not so frequently as he might be justified in doing because he fears to be looked upon as opposing progress. He knows that he can not give continuous hours of attention to any one lady when there are dozens of other patients equally entitled to his ministrations and he knows that even if he did spend the time, there is no surety that the results will be amnesic peace.

And when the newspapers and learned women who would lead us all into fields of grace and groves of endless beautitude attempt to show us what to do and make our patients demand that thing from us, they are reckoning without the cost and without ever having carried the responsibility of life and death. They are assailing the kind of intelligence that has its crucial training in the knowledge that every action has possibly fatal importance and significance if error occurs. That kind of intelligence the family doctor has and he knows how to use it. It is his verdict upon Twilight Sleep that will be the final one.

